# Hygiene in the East and in the West

**Published:** 1898-06-25

**Authors:** 


### Hygiene in the East and in the West.

The principles of hygiene are no doubt the same whether in the East or in the West, but the details, and the character of the difficulties to be overcome, differ greatly according to the country, and especially according to the people with whom we have to deal. This is strikingly brought home to us by the fact that only now are we attempting in the East to deal with a matter which, to Western views, would seem to touch the very foundations of sanitary science, namely, the old and world-wide practice of carrying water in skins. The problem of water supply in the West hinges principally on the purity of the source from which the water is derived, and we are apt to take for granted that, when we once have got the water, its distribution is a mere matter of taps and pipes. In India, however, it is otherwise, for there they are only just beginning to escape from ancient methods. Even where pipe water has been laid on by municipalities it is often carried to the houses in mussacks—the old leathern bottles which were in use before history began—so that the chances of pollution during distribution are quite as important as those arising from impurity of source.

In some of the stations in India the Government is getting rid of this old dirty method, so far as the troops are concerned, by laying pipes into the kitchens in the cantonments, and we may fairly hope that this measure will have the effect of lessening the typhoid fever which is such a curse to our troops in India. We must not, however, be too sanguine, for the habits of the natives are such as sometimes to defy the efforts of the sanitarian. Still the native does after all tend to do what comes the easiest, and if the water is brought actually into the kitchen in pipes, it is to be presumed that he will use it, although the average sweeper will generally somehow find a way for defiling even the purest and the cleanest things. In regard to mussacks, and their influence in spreading disease, our readers will remember the observations made a few years ago by Mr. Hankin as to their liability to become infected with cholera bacilli, and to go on imparting their infection to successive charges of water.

The bheestie, although employed for the special purpose of bringing water from a reputedly pure source, will never carry it farther than he can help; and thus it happens that, unless he is well looked after, he will now and again fill his mussack at the nearest tank, however polluted it may be, and once the mussack is infected, and the bacilli have taken root in its slimy interior, it will infect all the water which is put into it, however pure the source may be from which it comes. Hence the elimination of the mussack is a great step towards the prevention of the water-borne diseases from which India suffers. Again, however, we say we must not be too sanguine; for it is not the mussack itself that does the harm, but the native who misuses it, and the native is always with us—good, faithful fellow in his way, but steeped to the eyes in prejudice, and bad to move along sanitary lines. The native cook, with his peculiarly dirty ways, is responsible for much, and the fact that the cleaning of the cook-house is left to a sweeper is answerable perhaps for more, so that while the introduction of the pure tap-water into the kitchens is a great thing, the difficulties arising from native habits still remain. In the article on "Water-borne Diseases" in the "Twentieth Century Practice of Medicine," the following remarks appear in regard to cholera: "The phrase 'water-borne,' then, as applied to cholera, does not refer merely to the infection of great water supplies giving rise to vast epidemics, but has to do with cups and plates and cooking utensils, and the water in which they are washed; with salads and vegetables, and the water they are irrigated with in the garden and rinsed in the kitchen; and with the personal cleanliness of all who are concerned in the preparation, storing, and distribution of food or drink. In this sense nearly all cholera, at one part or another of its course from man to man, is water-borne." What applies to cholera applies equally to typhoid fever. But when we use the term in this wide sense we see clearly that the *rôle* of hygiene in regard to water supply, far from ending at the tap, is then only just beginning. The abolition of the mussack would be a step in the right direction, but even that is still far off, while the education of the native involves a multitude of steps, each of which is more difficult than the laying of many pipes into scores of kitchens.

